# Detrimental effect of Hypericum perforatum on ovarian functions

**DOI:** 10.4274/jtgga.galenos.2018.2018.0041

**Published:** 2019-05-28

**Authors:** Buket Demirci, Fadime Kahyaoğlu, Tolga Atakul, Mustafa Yılmaz, Yavuz Özoran

**Affiliations:** 1 Department of Medical Pharmacology, Adnan Menderes University Faculty of Medicine, Aydın, Turkey; 2 Department of Pathology Laboratory Tecniques, Avrasya University Vocational School of Health Services, Trabzon, Turkey; 3 Department of Obstetrics and Gynecology, Adnan Menderes University Faculty of Medicine, Aydın, Turkey; 4 Department of Medical Biochemistry, Adnan Menderes University Faculty of Medicine, Aydın, Turkey

**Keywords:** Anti-mullerian hormone, ovarian capacity, rational drug treatment, rat, St. John’s wort

## Abstract

**Objective:**

*Hypericum perforatum* is widely used for depression and distress treatment as an over-the-counter plant at any age. This study investigated the safety of *H. perforatum* on ovarian function and infertility.

**Material and Methods:**

*H. perforatum* was given to rats in two different dosages (100 and 300 mg/kg/day) with drinking water for four weeks. Half of the treatment groups were sacrificed at the end of the four-week intervention, the remainder was sacrificed after an additional four-week waiting period to see if there was reversibility. At the end of the experiment, blood samples and both ovarian tissues were obtained under anesthesia with ketamine and xylazine (50 mg/kg and 5 mg/kg, respectively).

**Results:**

Although primordial follicle numbers were not affected with a dose of 100 mg/kg, they were significantly decreased (28.6%) when the dose was tripled. Primary follicle numbers stayed the same, but secondary and tertiary follicles numbers were significantly dose-dependently decreased, and remained significantly low four weeks after the intervention. Anti-mullerian hormone (AMH) levels were not significantly different between the groups.

**Conclusion:**

*H. perforatum* treatment did not change serum levels of AMH because the primary follicle number did not decrease. However, the other follicle counts decreased in a dose-dependent manner and full recovery was not regained after four weeks. The detrimental effect of *H. perforatum* on primordial follicles should be taken into consideration because any woman using *H. perforatum* could also experience ovarian failure.

## Introduction

Although psychological distress by itself can cause psychosomatic infertility or decrease treatment success (1), the treatments of distress and depression with medicines and plants may also have a negative impact on ovarian function. There is a growing interest in plants and their derivatives, with an assumption that they are a safe method of treatment. Patients with infertility are increasingly using complementary and alternative medicines to support or replace medical fertility treatments (2). Extracts from the plant *Hypericum perforatum *(St John’s wort) have become an appealing alternative therapy to prescription serotonin-modulating drugs and are widely available to women of childbearing age (3,4).

There is little research on *H. perforatum* and its use in pregnancy, and its effect on fertility has not been established, even in European Medicines Agency guidelines (EMA) (5). Therefore, we evaluated impact of *H. perforatum* on ovarian tissue by counting follicles and assessing anti-mullerian hormone (AMH) levels.

## Material and Methods

### Chemicals and animals

*H. perforatum *(St. John’s Wort Herb Extract/SOLGAR, İstanbul, Turkey) was obtained from a local pharmacy store. Thirty-five 16-20–week-old female Wistar rats were obtained from the university and all experiments were performed according to the principles and guidelines of the University Animal Ethical Committee’s approval (HADYEK 64583101/2016/3). The “Principles of laboratory animal care” (NIH publication no. 86-23, revised 1985) and specific national laws were followed throughout the study. On the study day, the rats were randomly assigned to five groups of seven animals each.

### Experimental design

**Control group:** The rats in this group were allowed free access to tap water.

**Low-** ***H. perforatum*** **group:** The rats in this group were administered 100 mg/kg *H. perforatum* with drinking water for four weeks.

**Low-** ***H. perforatum*** **waiting group:** The rats in this group were administered 100 mg/kg *H. perforatum* with drinking water for four weeks, and then the rats received no medication and were sacrificed 4 weeks later.

**High-** ***H. perforatum*** **group:** The rats in this group were administered 300 mg/kg *H. perforatum* with drinking water for four weeks.

**High-** ***H. perforatum*** **group:** The rats in this group were administered 300 mg/kg *H. perforatum* with drinking water for four weeks, and then the rats received no medication and were sacrificed 4 weeks later.

The rats were weighed every monday and the doses of *H. perforatum* were adjusted for every cage. At the end of the experiment, under the anesthesia with ketamine and xylazine (50 mg/kg and 5 mg/kg, respectively), blood samples were obtained by cardiac puncture, centrifuged at 3000 rpm/4 °C, and sera were stored at -80 °C for AMH measurement. In addition, both ovaries were harvested and kept in 10% formalin.

The suggested daily dosage of *H. perforatum* for humans is 900 mg (or 15 mg/kg per day for a 60 kg human being); Rayburn et al. (3), calculated the rodent dosage as 180 mg/kg per day in their study. Considering that study, we wanted to apply two different doses of *H. perforatum* treatment to have a better understanding of dose effect and determined doses of 100 mg and 300 mg/kg (low and high). Additionally, we sacrificed some rats after a waiting period to determine if there was any reversibility of effect.

### Follicle counting

After routine tissue processing, the obtained samples were sliced in 5-micrometer thickness and evaluated under an optical microscope (Zeiss Primo star, Ankara, Turkey) with hematoxylin-eosin (H-E) staining. Oocyte classification was based on previous studies (6,7).

**Figure 1a-m:**
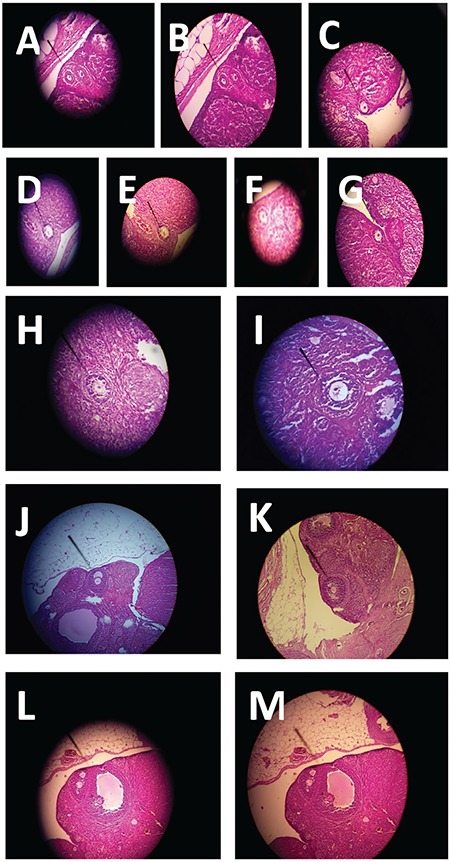
Pictures of primordial (a, b, c magnification ×10) and primer follicles (d, e, f ×10 magnification; g Magnification ×40), pictures of secondary (h, i magnification ×10) and tertiary follicles (j, k ×10 magnification; l, m magnification ×40)

Primordial follicle; follicles comprising a central oocyte and surrounding monolayer of flat squamous granulosa cells (Figure 1a-c).

Primary follicles; a central oocyte and surrounding monolayer of cubic granulosa cells or at least 3 cubic epithelial cells in monolayer granulosa cells (Figure 1d-g).

Secondary follicle (pre-antral follicle); follicles containing two or more layers of granulosa cells, without formed antrum follicles (Figure 1h, i).

Tertiary follicle (antral follicle, graff follicle); follicles containing two or more layers granulosa cells, with formed antrum follicles (Figure 1j-m).

### Measurement of serum rat AMH

The serum level of rat AMH was determined using a commercial enzyme-linked immunosorbent assay (ELISA) kits (rat ELISA kit, Sunred, Baoshan District, Shanghai, China). The rat AMH ELISA kit sensitivity was 0.101 ng/mL with a coefficient of variation of <5%. Procedures were performed according to manufacturer’s instructions.

### Data presentation and statistics

AMH levels and ovarian count evaluation was performed  using the Mann-Whitney U test. Data are presented as mean ± standard error mean, p values below 0.05 were considered significant. Follicle counts are also represented as percent changes.

## Results

The control groups’ number of primordial follicles number was 41.86±0.96, primary follicles: 12.29±0.42, secondary follicles: 9.00±0.66, and tertiary follicles: 23.29±0.81 (Table 1, Figure 1).

At the end of four weeks of low-dose *H. perforatum* treatment (100 mg/kg), the primordial follicles number increased by 2.03% (42.71±1.11), but decreased by 11.63% (10.86±0.55), 31.7% (6.14±0.40) (p<0.01), and 62.6% (8.71±0.61) (p<0.01) for primary, secondary, and tertiary follicles, respectively. Four weeks after ceasing *H. perforatum* treatment, the primordial follicle number (44.86±1.28) was still 7.1%, slightly higher than the control (41.86±0.96), the primary follicle number was nearly equal to the control value (12.43±0.37), the secondary follicle number was 65.1%, which was higher than the control (14.86±0.60) (p<0.01), and tertiary follicles had recovered mostly, but still remained 10.4% (20.86±0.96) (p<0.05) lower than the control value.

In the high-dose *H. perforatum* treatment group (300 mg/kg), the primordial follicle count dropped 29.0% (29.71±1.15) (p<0.01), and after the waiting period of four weeks the number was even lower 43.6% (23.57±0.84) (p<0.01). Although the primary follicle number did not change in both situations (11.57±0.65, 11.71±0.92), the number of secondary follicles did not change at the end of treatment (8.86±0.51), but declined 65.1% (3.14±0.60) (p<0.01) four weeks later. At the end of treatment, the decline of tertiary follicles was 87.7% (2.86±0.74) (p<0.01), and recovered to 9.29±0.68 but remained 60.1% (p<0.01) lower than control.

Serum AMH level of the three groups were not significantly different from each other (p>0.05); the control group’s AMH level was found as 4.39±0.47 ng/mL, the low-dose *H. perforatum* group was 4.63±0.63 ng/mL, and the high-dose *H. perforatum* group was 5.02±0.29 ng/mL.

## Discussion

Many researchers have focused on the beneficial anti-depressive effects of *H. perforatum*, and despite there being few reports about its harmful effects, unfortunately, this does not signify the plant’s safety, it shows a lack of rigorous research. This over-the-counter plant is available in many countries as an alternative treatment modality to anti-depressive medicines (5). The EMA determined in its guidelines the adult dosage is a total 900 mg/day (three-times 300 mg/day) for the treatment of depression (5). Unfortunately, it has been reported that it can be abused among adolescents who believe it can make them feel good (8), in this case, its limited upper dose has not been established due to its non-prescription use. Depression can affect people of any age and disturb fertility, and any woman could be exposed to *H. perforatum*, assuming that it is safe, either before being pregnant and even while pregnant. Therefore, this study has investigated harmful effects of *H. perforatum* on ovarian tissue in two different doses and evaluated reversibility and if there was any alteration on ovarian reserve.

In this study, 100 mg/kg doses of *H. perforatum* did not inhibit the primordial stage, but when the dose was tripled, these parts of the follicles were also affected. The primary follicle number remained the same in both doses; secondary and tertiary follicles numbers decreased in a dose-dependent manner and the ovarian cell count waiting did not recover after a four-week treatment cessation period. An earlier study showed that one-hour pretreatment of *H. perforatum* (0.6 mg/mL) resulted in zero penetration of hamster zona-free oocytes and significant denaturation of sperm DNA to decrease the sperm viability, leading to concerns that its use may lead to decreased fertility (9). When *H. perforatum* was given as 180 mg/kg to mice two weeks before mating, the authors found no specific concerns about the pregnancy rates of the treated and non-treated groups; however, the study was focused on pup development and the number of live pups per litter were not found different between the groups (3).

There are no toxicity reports with regard to exposure to *H. perforatum* on direct ovarian tissues in the literature, but it is possible to find some deleterious effects on other organs. Gregoretti et al. (10) gavaged rats during gestation and for 21 days during lactation at doses 100 or 1000 mg/kg and renal and hepatic damage was identified in the pups at both doses. We did not want to use 1000 mg/kg doses of *H. perforatum*, which seem very high, our aim was just to mimic real life and worked with lower-moderate doses as 100 mg/kg and 300 mg/kg, following Rayburn et al. (3) study.

We also searched reports of HP on hormonal status in the literature. One study evaluated *H. perforatum* (900 mg/kg) interaction with oral contraceptive therapy in sixteen healthy women. As the CPY3A4 enzyme was induced, which is well-known aspect of *H. perforatum*, the metabolism of these hormonal components was increased by approximately 25% and resulted in breakthrough bleeding, pre-ovulatory follicles and follicle exceeding (11). Thirty-six women aged 18-45 years with regular menstrual cycles who were diagnosed as having mild premenstrual syndrome were given *H. perforatum* (900 mg/day); in the follow-up, there were no differences in plasma follicle-stimulating hormone, luteinizing hormone, estradiol, progesterone, prolactin, and testosterone found compared with the non-treated group (4). Contrarily, hypo-prolactinemic activity has been reported in healthy male volunteers and plasma growth hormone levels increased due to dopaminergic action of *H. perforatum* (12). Plasma cortisol levels were significantly elevated in only four male volunteers out of twelve (12). Similarly, Di Carlo et al. (13) showed an inhibitory activity of *H. perforatum* (100 mg/kg) on prolactin production in male rats with 15 days’ treatment and suggested its clinical reflection might be luteal inadequacy, probably observed due to following pharmacologically-induced low prolactin secretion. An animal study on the hypothalamic-pituitary-adrenal axis (HPA) determined that two weeks’ treatment with hypericin (0.2 mg/kg) significantly down-regulated circulating plasma levels of ACTH and corticosterone; the authors suggested that this flavonoid of *H. perforatum* played an important role in the modulation of HPA axis function (14). Considering that stress and anxiety activate the HPA, and this activation can disturb the hormones of fertility (1), *H. perforatum* treatment sounds beneficial, but this approach requires detailed analysis. These previously published reports show that *H. perforatum* has the capability to interact with endocrine pathways; at some point, this might be helpful to explain the detrimental effect of *H. perforatum* on follicle growth. Interestingly, it was not possible to find a current study about *H. perforatum* and hormone profiles.

AMH is produced by the granulosa cells of early developing follicles and inhibits the transition from primordial follicles to the primary follicular stage. AMH can be measured in serum and has been shown to be proportional to the number of small antral follicles (15). It has been suggested that there is a strong positive correlation between serum AMH levels and antral follicle count; the use of AMH combined with antral follicle counts may improve ovarian reserve evaluation (16). In our study, we found no significant changes in AMH levels and also the primary follicle reserve remained stable with both doses of *H. perforatum*.

It has been clearly shown that *H. perforatum *treatment decreased follicle counts in a dose-dependent manner, especially primordial and tertiary pools. This study should help navigate future research on the hormonal aspect of the  effect of *H. perforatum*, which has to be investigated in detail. Any woman who takes *H. perforatum *could experience ovarian failure.

## Figures and Tables

**Table 1:**
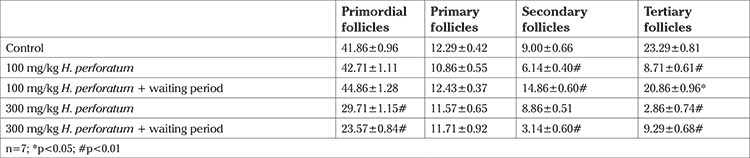
Ovarian follicles counting of all experimental groups
